# Incidence and pattern of injuries among residents of a rural area in South-Western Nigeria: a community-based study

**DOI:** 10.1186/1471-2458-7-246

**Published:** 2007-09-17

**Authors:** Omoniyi A Olawale, Eme T Owoaje

**Affiliations:** 1 Department of Community Medicine, University College Hospital, College of Medicine, University of Ibadan, Nigeria

## Abstract

**Background:**

Despite the high incidence of infectious diseases in developing countries, injuries still contribute significantly to the health burden. There are few reports of rural, community-based injury surveys in Nigeria. This study describes the incidence and pattern of injuries among the residents of a rural area in South-Western Nigeria.

**Methods:**

It was a community based cross-sectional study. Two of six census areas were randomly selected and all households in the two areas visited. Information on the sociodemographic characteristics, individual injury events and outcomes was obtained with a questionnaire. Data were analyzed using SPSS version 11.

**Results:**

Information was obtained on the 1,766 persons in 395 households. Fifty-nine injuries were recorded by 54 people, giving an injury incidence of 100 per 1,000 per year (95% CI = 91.4–106.9). Injury incidence among <30 years was 81.6 per 1,000 per year (95% CI = 62.3–83.1); and 126 per 1,000 per year (95% CI = 98.2–137.4) for those ≥ 30 years (p = 0.013). Injury incidence for females was 46 per 1,000 per year; and 159 per 1,000 per year (p = 0.000) for males. A significantly higher proportion of males (5%) sustained injury compared to females (2%) (p = 0.043). Falls and traffic injures, 15 (25%) each, were the leading causes of injury; followed by cuts/stabs 12 (21%), and blunt injuries, 9 (15%). Traffic injuries were the leading cause of injuries in all age groups except among the 5–14 years where falls were the leading cause of injury. In thirty-four (58%) of those injuries, treatment was at a hospital/health centre; while in two (3%), treatment was by untrained traditional practitioners. Thirty-nine (66%) of the injuries were fully recovered from, and 19 (32%) resulted in disability. There were 2 fatalities in the 5-year period, one (2%) within the study period.

**Conclusion:**

Injuries were common in Igbo-Ora, though resultant disability and fatality were low. Males and those aged ≥ 30 years had significantly higher proportions of the injured. Falls and traffic injuries were the most commonly reported injuries. Appropriate interventions to reduce the occurrences of injuries should be instituted by the local authorities. There is also need to educate the community members on how to prevent injuries.

## Background

Injury is recognized as a major health problem as well as a leading cause of death and disability globally [1,2], and in Africa. The associated morbidity, disability, socioeconomic losses, Gross National Product (GNP) and Disability Adjusted Life Year (DALY) losses have been documented by several studies [1-3]. Several workers have studied the causes, patterns, and outcomes of injury, and sociodemographic factors associated with injury [3-10]. However, few workers have described the pattern of injuries, and associated factors, in Nigeria. Moreover, majority of these studies have been hospital-based [10].

The mortality and economic losses imposed by morbidity resulting from injuries are largely preventable. However, the development of effective injury prevention efforts depends on reliable and detailed information on the incidence and pattern of injury. In developed countries, such data are available from vital statistics registers and health care records. However, such records are of limited value in developing countries. Many ill or injured persons in these countries never receive medical care from orthodox health facilities, and many deaths are not reported; making health records an incomplete source of data.

Injury as a research problem has also been largely ignored in developing countries(Tursz, 1986; Zwi, 1996; Krug et al, 2000). Not enough research has been done on injury as opposed to infectious diseases which constitute a major cause of morbidity and mortality in such countries. Moreover, recent progress in industrialization and use of vehicles, increased number of people living in crowded and unsafe urban settlements, coupled with inaccessible and unaffordable emergency health services, also contribute to the higher health burden of injury in these regions of the world.

Injury is thus a long-overlooked health problem that deserves study. This study was conducted to understand and describe the incidence and pattern of injuries in Igbo-Ora, a rural area in South-Western Nigeria.

## Methods

### Study design

It was a community-based, cross-sectional descriptive study conducted in April-May, 2005 in Igbo-Ora, the headquarters of Ibarapa Central Local Government Area, a rural area in Oyo State, South-Western Nigeria. Igbo-Ora has a population of about 60,000 and lies about 80 km away from Ibadan, the state capital. It is situated in the rain forest belt of the country. Majority of the residents are the native Yorubas, the other residents being made up of other nationals. The main occupations are farming and trading. Social infrastructure available include electricity, mobile telecommunications, several manually operated boreholes, tarred road network, a state general hospital and other health centres, primary and secondary schools. There is also a medium scale industry. Literacy rate is low, especially among the elderly. We, however, do not have exact figures.

The Ibarapa programme was initiated in 1963 as a joint effort of the Federal Government of Nigeria, the local traditional authorities, and the Department of Community Medicine, University College Hospital, University of Ibadan. It is a pilot scheme for developing locally relevant, and participatory community intervention programmes that can be sustained in the spirit of self-reliance, to serve as a source of data for community-based research for the department, and for the public health training of undergraduate and postgraduate students of the department and other affiliated health schools. To facilitate this, Igbo-Ora community, which is fairly homogenous, was divided, on geographical basis, into six census areas. This same division was employed for this study which was conducted as a student research under the Ibarapa programme.

The study population was all the people who had been residing in Igbo-Ora for at least 6 months prior to the study period.

### Sampling technique

Two out of the six census areas in Igbo-Ora were randomly selected, and all the households in these two census areas were visited. Since the whole community was fairly homogenous, the two randomly selected census areas can be expected to yield a representative sample.

### Ethical approval and informed consent

Ethical approval for the study was granted by the Ethics Committee of the Department of Community Medicine, University College Hospital, University of Ibadan, Ibadan, Nigeria – the university department administering the Ibarapa Programme; and the local authorities. Verbal informed consent was obtained from the head of the households and individual adults. For minors, verbal informed consent was obtained from the parents/guardians. Appropriate rapport was established before administering the questionnaire.

### Data collection

The data were collected by the use of a structured questionnaire adapted (and modified) from an Ugandan survey by Kobusingye et al in 1998 [6]. The questionnaire had two sections:

#### Household characteristics

Number of persons in the household, type of dwelling, type of energy sources used, and demographic information about each member of the household, whether or not they have suffered any injury in the last 4 months. We also included mortality due to injury in the last 5 years.

#### Individual injury event

Collection of detailed information on each injury events: the cause of injury, injury sustained, part of the body affected and outcomes of injury. [see Additional file 1: "Study questionnaire" for the questionnaire].

The questionnaire was interviewer-administered, structured in English to ensure accuracy. The questionnaires were pre-tested at Idere, a similar environment about six kilometres away from the study area; and necessary adjustments were made to the questionnaire. The interviewers were pre-trained on administering the questionnaire to ensure uniformity, and accurate use of the local language in a culturally sensitive manner.

Although the unit of sampling was a household, the unit of analysis was an injury sustained in the 4-month period preceding the study. Injury was defined, in the local language, as any type of injury on any part of the body, in the last 4 months prior to the study; regardless of severity and outcome. Common examples of injury and injury causes were mentioned to ensure clarity. Disability was defined as loss of at least one day of work/school due to the direct effect of an injury sustained within the four-month period preceding the study. A major injury was one that led to at least 30 days lost and a minor injury less than 30 days. The recall period was four months. We had earlier wanted to use a recall period of six months, as in the Uganda study. However, preliminary work showed technical difficulties: most of the respondents were not formally educated and found it difficult to count/register months of the year. We, therefore, decided to make the start of the recall period the beginning of the year, since that was easier for respondents to register. The respondent was the head of the household or any other responsible adult. Information about individual injuries was obtained from the injured person. If the injured person was absent or a child, the information was obtained from the respondent (head of household/responsible adult).

The data were collected in an anonymous manner, without any names or other identifying information. The data were recorded blindly and the materials kept in a locked up room for disposal later – as is done periodically.

### Statistical analysis

Data collected were entered and analyzed using SPSS version 11. Simple descriptives and frequencies were used. Statistical association was investigated using the chi-square test.

## Results

Three hundred and ninety-five respondents in 395 households were interviewed and information was obtained on the 1,766 persons residing in these households. The response rate was 82% for households, and 86% for individuals within the households. Majority, 212 (54%), of the respondents were in the 15–49 age group. Females, 241, made up 61% of the respondents.

The ages of the 1,766 persons in the population ranged from 2 months to 85 years with a mean age of 40 ± 17 years. The 15–49 age group had the highest population with 811 (46%). Eight hundred and forty-nine (48.1%) were males; while 917 (51.9%) were females [see Table 1].

**Table 1:** Study population by age and sex

Age group(years)	Total		Male		Female	
	
	No	%	No	%	No	%
0–4	210	12	96	11	114	13
5–14	415	23	212	25	203	22
15–49	8111	46	397	47	414	45
50+	330	19	144	17	186	20
**Total**	**1766**	**100**	**849**	**100**	**917**	**100**

Fifty-nine injuries were recorded in the 4-month period, by 54 people; one person had four injuries and two had two injuries each within the study period. The annual injury incidence was 100 per 1,000 per year (95% CI = 91.4–106.9). The number of injuries that led to the loss of at least a day of work/school was 44, with an incidence of 75 per 1,000 per year.

The ages of the injured people ranged from 3 years to 65 years; with a mean of 30 ± 15 years. The age group 15–49 had the highest frequency (34, 58%) of those injured. The injury incidence in those below 30 years was 81.6 per 1,000 per year (95% CI = 62.3–83.1); and 126 per 1,000 per year in the ≥ 30 years age bracket (95% CI = 98.2–137.4) (p = 0.013).

Males accounted for 45 (76%) of the injuries, with an annual injury incidence of 159 per 1,000 per year; while the females accounted for 14 (24%) with an annual injury incidence of 46 per 1,000 per year [see Table 2]. A significantly higher proportion of injuries were sustained by males (5%) compared to the females (2%) (p = 0.043).

**Table 2:** Injury cause by sex

Injury cause	Total		Male		Female	
	
	No	%	No	%	No	%
Traffic	15	25	10	22	5	36
Falls	15	25	9	20	6	43
Cuts/stabs	12	21	11	24	1	7
Blunt	9	15	9	20	1	7
Burns	3	5	3	6	0	0
Others	5	9	4	8	1	7
**Total**	**59**	**100**	**45**	**100**	**14**	**100**

Students had the highest frequency of injuries, 18 (31%); injuries were least among the unemployed, 5 (8%) (not shown in table).

### Causes of injury

Falls and traffic injures, 15 (25%) each, were the leading causes of injury; followed by cuts/stabs 12 (21%), and blunt injuries, 9 (15%). Among males, cuts/stabs, 11 (24%), were the leading cause of injury; followed by traffic injuries,10 (22%). Among the females, falls, 6 (43%), were the leading cause followed by traffic injuries, 5 (36%). In all types of injuries, there was male preponderance [see Table 2].

**Figure 1:**
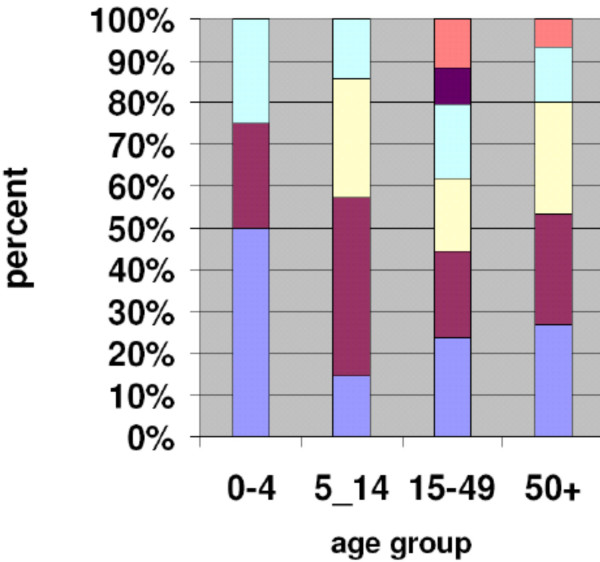
Injury cause by age group.

Traffic injuries were the leading cause of injuries in all age groups except among the 5–14 years where falls were the leading cause of injury [see Figure 1].

In 32 (54%) of the injuries, the lower extremities were affected, followed by the trunk, 14 (24%) and upper limbs, 8 (14%). Majority, 19 (32%), of the injuries occurred at home; followed by those on the road in town, 16 (27%), and farm, 14 (24%).

Forty-eight (81%) of the injuries were unintentional. Of the 11 intentional injuries, assault was most common, 6 (55%). Of the 59 injuries, 34 (58%) were treated at a hospital/health centre while two (3%) were managed by an untrained practitioner. Sixteen (47%) of injuries for which treatment was sought at a hospital/health centre required admission.

Fifty-one (86%) of the injuries were minor (led to a loss of less than 30 days of work/school). Only one (2%) of the injuries led to loss of more than 30 days of work/school by the caregiver.

### Injury outcomes

As at the time of the study, 39 (66%) of the injuries were fully recovered from, 19 (32%) led to disability. Two injury fatalities were recorded in the 5-year period preceding the study, one (2%) within the four-month study period [see Table 3].

**Table 3:** Injury outcomes by sex

Injury outcome	Total		Male		Female	
	
	No	%	No	%	No	%
Full recovery	39	66	31	69	8	57
Disability	19	32	14	31	5	36
Death	1	2	0	0	1	7
**Total**	**59**	**100**	**45**	**100**	**14**	**100**

## Discussion

Our study revealed an overall injury incidence of 100 per 1,000 per year, higher than comparable studies in Pakistan (41.3 per 1,000 per year) [3], and Tanzania (32.7 per 1,000 per year) [11]. The authors initially thought this could be explained by the all-inclusive definition of injury in the study to include all injuries regardless of severity, as against other studies in which only injuries that led to treatment in the hospital or loss of at least a day's work were recorded. However, the incidence of injuries leading to the loss of at least a day of work/school (75 per 1,000 per year) was still relatively high compared to other studies. This may be explained by the fact that, though Igbo-Ora is a rural area, it is bordered by bigger towns; and a number of interstate highways pass through it, including a trans-border smuggling route. There has also been a recent increase in the number of vehicles, especially motorcycles, in Igbo-Ora. Many of the motorcycle riders are neither formally trained nor licensed to ride motorcycles, and drive recklessly. These factors may account for high incidence of injuries, of which majority are traffic-related.

The mean age of the injured population (30 years ± 15 years) was similar to those in similar rural community-based injury surveys in Pakistan [3], and Tanzania [11], and this may be explained by the fact that a large proportion of the population is in the 15–49 years age bracket. It also underscores the economic impact of these injuries since they affect the productive age group.

The male preponderance among those injured agrees with the WHO data and the studies of Abdul Gaffhar et al (2004) [3], Mock et al (1997) [7], and Moshiro et al (2004) [11]. In all injury types, there were more males injured than females, similar to other study findings.

While our finding that traffic injuries and falls were most common agreed in part with the WHO global report that traffic injuries were most common, it differed from rural community surveys in Uganda and Ghana where it was drowning and agricultural injuries respectively. Our finding seemed to resemble more the situation in the urban areas in these studies, where traffic injuries were most common. This may be explained by the recent increase in number of motorcycles in Igbo-Ora. Motorcycle injuries accounted for a majority of the traffic injuries in Igbo-Ora, a situation earlier reported by Mock et al in Ghana (1997), and Moshiro et al in Tanzania (2004). The main road in Igbo-Ora is also along the car-smugglers' route and could explain the increased risk and occurrence of traffic injuries.

The occurrence of most injuries in the home and on the road in town agrees with the report of Kobusingye et al in 2001. The situation in Tanzania was slightly different, however, with more injuries occurring outside the home than within the home. That most of the injuries were unintentional was a finding similar to findings in other rural community-based injury surveys.

Our survey showed that the disability and fatality resulting from injury among Igbo-Ora residents were low, with most of the injuries being fully recovered from; a finding similar to the Uganda study.

The health-seeking behaviour of Igbo-Ora residents was good. Of those injured, more than half (58%) sought treatment in a hospital/health centre. This was in sharp contrast to the study by Edet et al [8] in the Idikan neighbourhood of Ibadan where less than one percent of those injured were treated in orthodox health facilities. Due to the low literacy rates and poverty, orthodox care accounts for minority of healthcare received in most rural areas of Nigeria. This unusual observation could be explained by the 42-year presence of the Ibarapa Programme in Igbo-Ora, which may have positively modified the health-seeking behaviour of the residents through various health education programmes and subsidized health care delivery.

Detailed demographic data are not available, but age and gender distributions of Igbo-Ora are comparable to the national data available from the 1991 census- which was the latest at the time of the study. Therefore, the results of this study can be reasonably expected to afford some cautious generalization to other rural areas in Nigeria. However, Nigeria is a very populous and heterogeneous country and this may limit the generalizability of the results. It however, provides vital preliminary information needed for planning local injury prevention programmes in Igbo-Ora and comparable rural areas in the country, and provides a stimulant for more extensive nationwide studies that compare rural and urban areas and investigate risk factors associated with injury incidence, causes and outcomes.

## Limitations

1. Self reporting: the accuracy of respondent's answer on the occurrence of injury events or on duration of resultant disability cannot be independently verified. That is, the answers to questions about nature of injury for any responses received could not be verified. Injury caused by sexual violence or domestic violence are likely to be underreported, since respondents do not want to disclose the fact that they have been victims.

2. Recall: there is a tendency for respondents to report events occurring outside the study period as if they had occurred within it, and vice versa. It was also likely that details of the injury could be lost due to memory-decay, thus a recall bias. The short recall period of 4 months should minimize this.

3. Proxy respondents: the use of the head of the household to recall all injuries sustained by the household predisposes to underreporting. If he/she has to respond for more than one person, the tendency for inaccurate injury reporting is increased.

4. Language difficulty: defining injury and disability in a culturally acceptable way could be difficult and may have affected the quality of data we got. To minimize this, we trained the personnel to administer the questionnaire using the local language in a culturally acceptable way.

We feel these limitations were minimized such that they should not significantly affect the quality of the results.

## Conclusion

Injuries have a relatively high incidence in Igbo-Ora compared to some other communities. Though the injuries do not have high mortality or disability rates, they predominantly affect the productive age group of the population. Males and those aged ≥ 30 years accounted for significantly higher proportions of the injuries.

Falls and traffic injuries were the leading causes of injury; with traffic injuries being the leading cause among males, and falls among females. The residents of Igbo-Ora showed good health-seeking behaviour.

This study in Igbo-Ora describes the incidence and patterns of injuries and associated socio-demographic factors. While this study is important for planning interventions to reduce injuries in Igbo- Ora and similar environments, there is a need for more detailed studies that compare rural and urban areas, and investigate risk factors associated with injury incidence, causes and outcomes; and studies on sensitive events such as assault and domestic violence. It is hoped that this study will stimulate the conduct of other studies all over the country to give a national picture.

## Competing interests

The author(s) declare that they have no competing interests.

## Authors' contributions

OA participated in the study conception, study design, data acquisition, analysis and drafting of the manuscript. EO participated in the study conception, study design, analysis and drafting of the manuscript. Both authors read and approved the final manuscript.

## Pre-publication history

The pre-publication history for this paper can be accessed here:



## Supplementary Material

Additional file 1 questionnaire. The document shows the instrument used to collect the data presented in the article. Click here for file
