# Supplementary material for: Incidence and pattern of injuries among residents of a rural area in South-Western Nigeria: a community-based study
Source: BMC Public Health. 2007 Sep 17;7:246. doi: 10.1186/1471-2458-7-246 (PMC2169237; doi:10.1186/1471-2458-7-246)
Supplement: Additional file 1 — questionnaire. The document shows the instrument used to collect the data presented in the article. [file 1471-2458-7-246-S1.doc]

QUESTIONNAIRE

**Incideance and pattern of injuries among residents of a rural area in South western Nigeria**

**A. Home environment and occupant demographic information**

1. Interviewer name _________ Date of interview _____________

We are going to talk about the following injury causes:

1. Traffic 2. Fall 3. Burn 4. Gun shot 5. Stab

6. Blunt injury 7. Poisoning 8. Drowning

9. Dog, snake or other animal bite 10. Other causes

When we talk, please remember to include all of these causes, both for injuries that happened on purpose (intentional) and those that happened by accident (unintentional).

2. ID number for household ___________ Zone _________________________

3. a) Type of dwelling 1. Temporary 2. Semi-permanent 3. Permanent ________

b) Number of habitable rooms (including kitchen) _________________________

4. How long have you been living here?_______________years ______________

months

5. Status in home 1. Head of household 2. Other (specify) ___________________

6. What is the most common cooking facility you use? (choose only one)

1. Electricity 2. Gas 3. Paraffi n 4. Coal/charcoal

5. Wood 6. Other (specify) ______________________________________

7. What is your primary source for lighting? (choose only one)

1. Electricity 2. Gas 3. Paraffi n lamp 4. Candles

5. Other (specify) _____________________________________**____________**

8. The accuracy of the following table is crucial for this survey. Please follow the steps

carefully.

List all people currently in household AND also those who have died of injury in the

last 5 years.

**Record the number of injury events for each individual. If an individual has**

**no event place a 0 in that cell so that all cells are filled. Some households will**

**have more than one injury form and some individuals will require more than**

**one injury form as well.**

| **Individual no.** | **Gender(M/F)** | **Present age or death age(years)** | **Number of injury events** | | |
| --- | --- | --- | --- | --- | --- |
|  |  |  |
| #1.  Respondent |  |  |  |  |  |
| #2. |  |  |  |  |  |
| #3. |  |  |  |  |  |
| #4. |  |  |  |  |  |
| #5. |  |  |  |  |  |
| #6. |  |  |  |  |  |
| #7 |  |  |  |  |  |
| #8. |  |  |  |  |  |

NOW GO TO SECTION B.

FILL OUT A SEPARATE FORM FOR:

• EACH DEATH IN THE LAST 5 YEARS

• EACH INJURY WITH DISABILITY IN THE LAST 4 MONTHS

• EACH INJURY WITH RECOVERY IN THE LAST 4 MONTHS.

**B. Individual injury event form**

DETAILED INJURY INFORMATION FOR ALL DEAD, DISABLED, OR INDIVIDUALS INJURED.

THERE SHOULD BE ONE FORM FOR EACH DEATH IN THE LAST 5 YEARS, ALL CURRENT

DISABILITIES AND FOR RECOVERED INJURIES IN THE LAST 4 MONTHS.

*Some people may have more than one form.*

Check one from the box:

| □Death (last 5 years) | □Disability (last 4 months) | □Recovered injury (last 4 months) |
| --- | --- | --- |

1. Household ID number _____________ Individual number __________ (*from table*)

Total number of injury events for this person in the last 4 months ________________

2. From the list below, what caused that death, disability or injury checked in the box

above? __________________________________________________________

1. Traffic 2. Unintentional fall 3. Burn 4. Gun shot 5. Stab

6. Blunt injury 7. Poisoning 8. Drowning

9. Dog, snake or other animal bite 10. Landmine 11. Other causes

3. Did the death, disability or recovered injury occur in the last 4 months? _______

1. Yes 2. No

4. Did the event happen in the study area? _______

1. Yes 2. No

5. Date of injury leading to death or disability _____ year _____month

6. Age (at death, disability, or injury) _____ years _____months

7. Occupation of person _______

1. Peasant farmer 2. Student/pupil 3. Housewife

4. Pre-school child/baby 5. Civil servant/private employee

6. Causal labourer 7. Driver/turn boy/conductor

8. Small business owner 9. Large business owner

10. Unemployed 11. Other (specify) ___**_______________________**

8. Where did the injury happen? ________

1. Home 2. Farm 3. Industry

4. Bar/disco 5. Road in town 6. Road in country

7. Public or offi ce building 8. School 9. Sport

10. Cinema or theatre 11. Other (specify) __________________________

9. Was the injury intentional or unintentional? _______

1. Unintentional

2. Intentional

a. Assault/homicide b. Abuse c. Self-infl icted d. Legal intervention

e. Terrorism/war

3. Undetermined

10. How did it happen? ________________________________________________

________________________________________________________________

11. Who initially tried to help the deceased, disabled or injured person in this event?___

1. Bystander 2. Friend/family 3. Police

4. Ambulance 5. Fire brigade 6. No help

7. Other (specify) ______________________ 8. Unknown

12. Did he/she seek treatment outside the household? ________________________

1. Yes 2. No

13. If yes, where did he/she seek treatment? _______

1. Hospital 2. Health centre 3. Clinic

4. Community health worker 5. Untrained practitioner or drug store

6. Traditional practitioner 7. Other (specify) ___________________________

14. Was he/she admitted? _______

1. Yes 2. No 3. Unknown

16. In the case of death where did he/she die?

1. At the injury scene 2. In hospital 3. Elsewhere (specify) ____________

17. When did the injury happen?

Day: ___________ 1. Weekday 2. Weekend

Time: __________ 1. Day light 2. After dark

18. How many days of work or school were lost by the injured person? ___________

1. None 2. Less than one week

3. From one week to a month 4. More than one month

19. How many days of work or school were lost by the household caregiver? _______

1. None 2. Less than one week

3. From one week to a month 4. More than one month

20. Did the household have to borrow money to take care of the injured person?

1. Yes 2. No 3. Unknown
